# Genes associated with hot defensive bee ball in the Japanese honeybee, *Apis cerana japonica*

**DOI:** 10.1186/s12862-022-01989-9

**Published:** 2022-03-16

**Authors:** Takahiro Kamioka, Hiromu C. Suzuki, Atsushi Ugajin, Yuta Yamaguchi, Masakazu Nishimura, Tetsuhiko Sasaki, Masato Ono, Masakado Kawata

**Affiliations:** 1 Graduate School of Life Sciences, Tohoku University, 6-3, Aramaki Aza Aoba, Aoba-ku, Sendai, 980-8578 Japan; 2 Department of Integrative Biology, University of California-Berkeley, Berkeley, CA 94720 USA; 3 JT Biohistory Research Hall, Takatsuki, Japan; 4 Graduate School of Agriculture, Tamagawa University, Machida, Japan; 5 Research Institute, Honeybee Science Research Center, Tamagawa University, Machida, Japan

**Keywords:** *Apis cerana japonica*, Hot defensive bee ball, RNA-seq, Differential gene expression, Thermal sensitivity, Rhodopsin

## Abstract

**Background:**

The Japanese honeybee, *Apis cerana japonica*, shows a specific defensive behavior, known as a “hot defensive bee ball,” used against the giant hornet, *Vespa mandarinia*. Hundreds of honeybee workers surround a hornet and make a “bee ball” during this behavior. They maintain the ball for around 30 min, and its core temperature can reach 46. Although various studies have been conducted on the characteristics of this behavior, its molecular mechanism has yet to be elucidated. Here, we performed a comprehensive transcriptomic analysis to detect candidate genes related to balling behavior.

**Results:**

The expression levels of differentially expressed genes (DEGs) in the brain, flight muscle, and fat body were evaluated during ball formation and incubation at 46 °C. The DEGs detected during ball formation, but not in response to heat, were considered important for ball formation. The expression of genes related to rhodopsin signaling were increased in all tissues during ball formation. DEGs detected in one or two tissues during ball formation were also identified.

**Conclusions:**

Given that rhodopsin is involved in temperature sensing in *Drosophila*, the rhodopsin-related DEGs in *A. cerana japonica* may be involved in temperature sensing specifically during ball formation.

**Supplementary Information:**

The online version contains supplementary material available at 10.1186/s12862-022-01989-9.

## Background

Temperature is one of the abiotic factors that affects insects. Given that climate change may affect the distribution of organisms [54], it is essential to understand the thermal adaptation mechanisms of insects. Exceptionally for insects, two species of honeybee, *Apis mellifera* and *Apis cerana*, inhabit regions with a wide temperature range from the tropical to the temperate zones [10]. Honeybees may have adapted to living in such a wide range of temperatures by acquiring temperature regulation abilities. Therefore, studies on honeybee thermoregulation are important for achieving a better understanding of insect temperature adaptation.

The body temperature of insects is controlled by the heat production via flight muscle [23–25] and by selecting the optimal temperature via temperature sensors [33, 61]. Honeybees maintain their nest temperature at 33–36 °C, the optimal temperature range for larval growth [15, 60, 74]. Nest temperature is maintained by worker bees that regulate the heat production using their flight muscles [32, 64]. Thus, nest temperature control requires heat regulation via both flight muscles and temperature sensing. Although several studies have investigated the molecular mechanism of temperature sensing in insects [7, 21, 47, 58, 61], the molecular mechanism underlying the heat regulation system in honeybees has yet to be clarified.

*Apis cerana,* a native species of Asia, has different characteristics from *A. mellifera*, such as being resistant to certain diseases [55, 76]. *A. cerana* also shows different thermal characteristics from that of *A. mellifera* such as a high heat production capacity [15] and a high activity level at low temperature [1, 72]. Thus, *A. cerana* may have different thermoregulation abilities than *A. mellifera*.

**Fig. 1:**
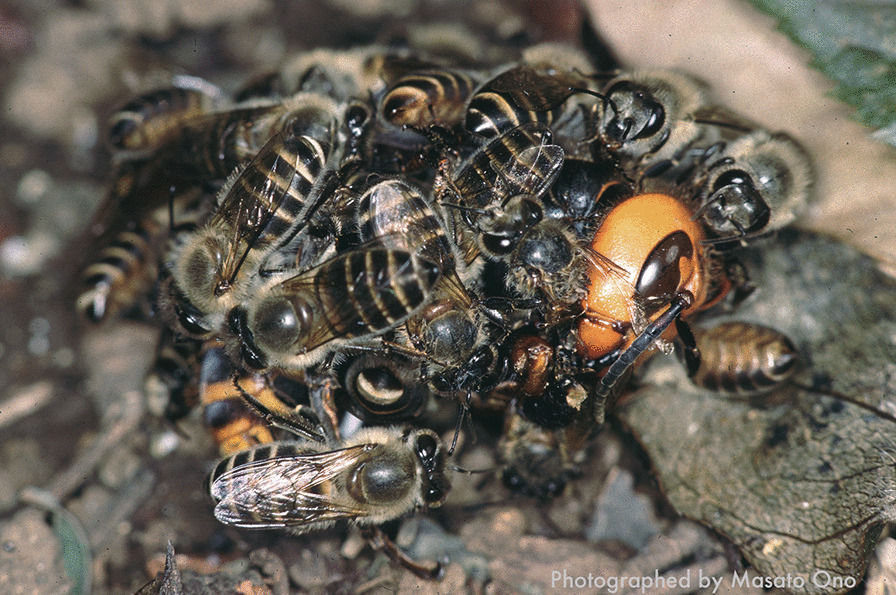
Hot defensive bee ball used against hornets by the Japanese honeybee, *A. cerana japonica* (Photographed by Masato Ono)

Additionally, *A. cerana* exhibits unique anti-predator behavior against hornets, one of the primary predators of honeybees in Asia [49], which involves precise temperature regulation [50, 51, 65]. In Japan, the giant hornet, *Vespa mandarinia*, attacks the honeybee nests *en masse* in autumn. Indeed, *V. mandarina* frequently destroys entire colonies of *A. mellifer*a [39], which was introduced to Japan only about 150 years ago (http://www.beekeeping.or.jp/beekeeping/history/japan) and does not have effective countermeasures against the hornets. By contrast, *A. cerana* (Eastern honeybee) displays a collective defensive behavior, first reported in the Japanese honeybee, *A. cerana japonica*. This behavior is known as a “hot defensive bee ball” because honeybee workers can kill hornets by surrounding them and producing the heat from their flight muscles (Fig. 1). Nest defense of *A. cerana japonica* using hot defensive bee ball consists of multiple steps [51]. First, when an individual *V. mandarinia* attaches markings to attract its nestmates toward the nest of honeybees, worker bees warm their flight muscles to prepare for the hot defensive bee ball. When *V. mandarinia* enters the nest, approximately 500 worker bees surround the hornet. These workers rapidly raise the temperature inside the ball, which can reach 46 °C, higher than the lethal temperature of the hornet, and they maintain the ball for around 30 min [51, 65, 66]. Using this balling behavior, honeybees can effectively kill the hornet while many workers survive [29, 51, 65], although the exposure to high heat during the bee ball affects the viability of *A. cerana japonica* [77]. Thus, the hot defensive bee ball is a specific thermoregulatory behavior that produces and maintains a dangerous temperature even for this species.

Although *A. mellifera* also makes bee balls to defend against hornets [5, 26, 41, 48, 50, 53], the hornet-killing efficiency of their behavior is lower than that of *A. cerana japonica* [2]. This low efficiency may be due to the lower temperature produced within the *A. mellifera* bee ball [29] and/or the higher mortality of workers during balling [69]. This comparison suggests that high predation pressure from hornets may have led to the refinement of balling behavior in *A. cerana japonica*.

The formation of the hot defensive bee ball of *A. cerana japonica* is a multifaceted process involving thermoregulation near the sublethal temperature, which provides a rare opportunity to elucidate the molecular basis of thermoregulation in honeybees. However, despite multiple studies into the characteristics of bee balling, few have attempted to clarify its molecular mechanism, except for one study that investigated neural activity during balling behavior [71].

Therefore, in this study, we performed comprehensive gene expression analysis using RNA sequencing (RNA-seq) to identify candidate genes related to balling behavior in *A. cerana japonica*. We identified genes for which expression levels changed during the bee balling process in the brain, fat body, and flight muscle, respectively.

## Results

### Global gene expression patterns

**Fig. 2:**
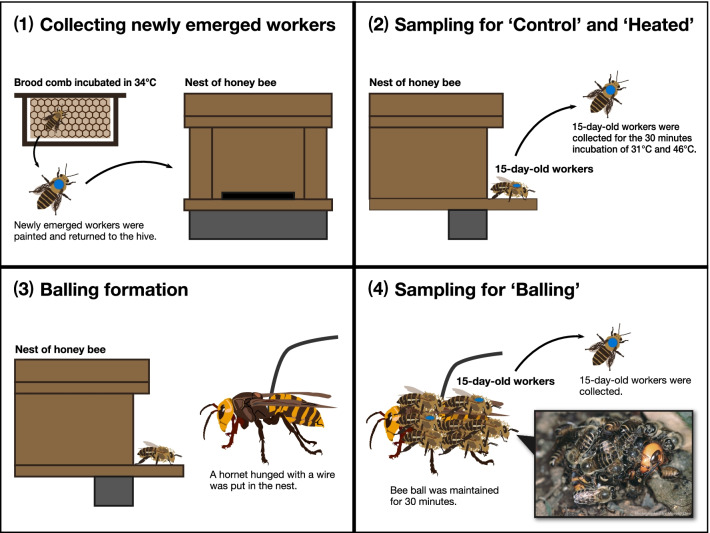
Schematic diagram of RNA-seq sampling. (1) In the first step of the experiment, to align the age of honeybee workers, workers newly emerged from a brood comb that was incubated at 34 °C were marked and returned to the nest. When marked workers reached 15 days old, they were collected for use in the following treatments. (2) Before the balling experiment, 15-day-old workers for “control” and “heated” groups were collected from the nest for the respective temperature treatments. (3) A hornet hung by a wire was placed into the bee nest, causing a bee ball to form. (4) At 30 min after the formation of the bee ball, the bees that participated in the bee balling were collected (“balling”)

**Fig. 3:**
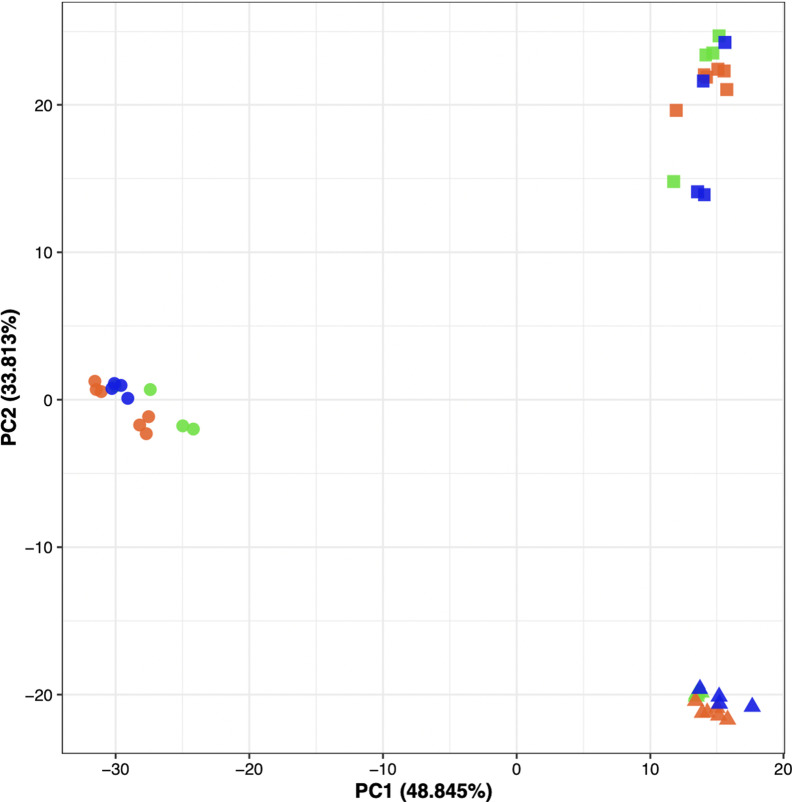
PCA plot for the gene expression patterns in *A. cerana japonica*. Each dot indicates the gene expression profile of a single bee for the brain, fat body, and flight muscles. The horizontal and vertical axes represent PC1 and PC2, respectively. Numbers in the parentheses show the proportion of the variance explained by PC1 and PC2. The shapes of the dots represent the organs (circle = brain; triangle = fat body; square = flight muscle) and the colors indicate the experimental treatments (green = “balling”; blue = “heated”; orange = “control”)

Worker bees after bee ball formation (“balling”, n = 4), short 46 ˚C incubation (“heated”, n = 4), or normal incubation (“control”, n = 6) were sampled for RNA extraction and the subsequent sequencing (Fig. 2; see Methods for the detailed sampling procedure). RNA-seq produced 1,509,857,212 reads in total from three tissues (i.e., the brain, fat body, flight muscle) of 14 *A. cerana japonica* individuals (Table 1). The maximum and minimum number of reads was 43,811,508 and 28,303,868, respectively. Total length of reads was 190,309,614 bp. These reads yielded 218,202 contigs after de novo assembly using Trinity (Table 1). The reciprocal BLAST search narrowed these down to 10,712 contigs that represented orthologs of *A. mellifera*. We used this gene set as a reference for short-read mapping to calculate FPKM using RSEM. The RNA-seq count data are listed in Additional file 1, and FPKM values are listed in Additional file 2. Approximately 90.95% of total reads were aligned onto the reference contigs (Table 1); the maximum and minimum rates were 92.73% and 89.23%, respectively. Principal component analysis (PCA) using the FPKM data showed that expression patterns were tightly clustered by tissues, not by experimental treatments (Fig. 3). The PC1 axis separated the brain samples from the fat body and the flight muscle, whereas PC2 separated all organs equally (Fig. 3). The FPKM density distribution and violin plots also showed that gene expression profiles were similar among treatments but differed among tissues (Additional file 5: Fig. S1).

**Table 1:** Summary of de novo assembly of RNA-seq reads of *A. cerana japonica*

Assembly assessment parameter	
Total number of reads	1,509,857,212
Maximum number of reads	43,811,508
Minimum number of reads	28,303,868
Total length	190,309,614
No. contigs	218,202
Mean contig length	872.2
Maximum length	25,150
Minimum length	201
Mean mapping rate	90.95%
Maximum mapping rate	92.73%
Minimum mapping rate	89.23%
No. *A mellifera* ortholog	10,172

### DEGs specific to balling behavior

**Fig. 4:**
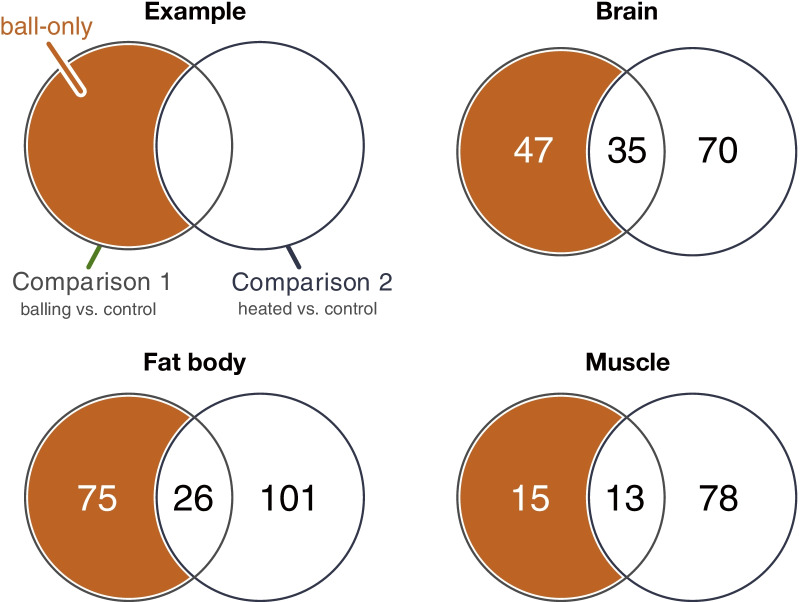
Numbers of DEGs detected in the brain, fat body, and flight muscle. Venn diagram indicates the overlaps of DEGs between Comparison 1 (“balling” vs. “control,” left circle) and Comparison 2 (“heated” vs “control,” right circle). Regions colored orange highlight the group of DEGs specifically observed during the bee ball formation (i.e., “ball-only” group, including DEGs detected only in Comparison 1)

**Fig. 5:**
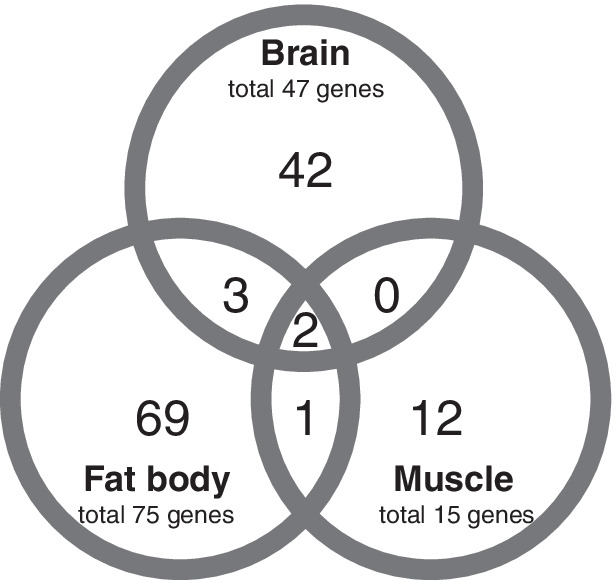
Numbers of “ball-only” DEGs in each tissue

Differentially expressed genes (DEGs) were detected among the three experimental treatments using the TCC package [67]. The pattern of DEG is shown in the MA plot (Additional file 6: Fig. S2). The numbers of DEGs in Comparison 1 (“balling” vs. “control”) were 82, 101, and 28 in the brain, fat body, and flight muscle, respectively (Fig. 4 and Additional file 7: Fig. S3a). The numbers of DEGs in Comparison 2 (“heated” vs. “control”) were 106, 127, and 91, respectively (Fig. 4 and Additional file 7: Fig. S3b). We defined DEGs from Comparison 1 after removing the overlapped DEGs with Comparison 2 as genes related to the bee ball formation (“ball-only”). In this group, 47, 75, and 15 DEGs were identified in the brain, fat body, and flight muscle, respectively (Fig. 5). A complete list of DEGs is provided in Additional file 3.

Surprisingly, two genes stood out as “ball-only” DEGs that are common across all tissues: *rhodopsin long wavelength* and *arrestin 2* (Additional file 3). These genes were upregulated in all tissues in the “balling” treatment (Additional file 3).

In the brain, genes associated with rhodopsin signal transduction were upregulated, such as *1-phosphatidylinositol 4,5-bisphosphate phosphodiesterase* (*PLC*)*, arrestin 1*, and *carotenoid isomerooxygenase* (*ninaB*). The gene expression of *nuclear receptor subfamily 4 group A member 2* (*NR4A2*) and *atrial natriuretic peptide receptor 1* (*NPR1*), which are related to dopamine metabolism, increased and decreased, respectively. Furthermore, the expression of *15-hdroxyprostaglandin dehydrogenase* (*HPGD*), related to inflammation, increased, as did the expression of *malate dehydrogenase* and *NADH dehydrogenase subunit 5*, which are associated with glucose metabolism.

In the fat body, gene expression of three *Tret1* genes related to trehalose transport increased, whereas the expression of *ACC* associated with fatty acid synthesis decreased. Several HSP genes, such as *HSP90-alpha* and *Hsc70-4*, were upregulated, whereas the expression of some immunity-related genes, such as *abaecin-like*, *hymenoptaecin*, and *defensin-1,* were downregulated.

In the flight muscle, the expression of genes that encode endochitinase and cuticle protein, which are involved in exoskeleton formation, increased and decreased, respectively.

### Pathway analysis and GO analysis

We also performed pathway analysis to reveal the overall trend of DEGs. The longevity regulating pathway was enriched in most of the categories we tested (Table 2 and Additional file 4: Table S1). Phototransduction, inositol phosphate metabolism, and phosphatidylinositol signaling were enriched in the "ball-only” category (Table 2). Protein processing in the endoplasmic reticulum and spliceosome activity were involved in Comparisons 1 and 2 (Table 2 and Additional file 4: Table S1).

**Table 2:** Result of pathway analysis in “ball-only” group

Organ	KEGG pathway	ID	Number of Genes	Corrected P-value
Brain	AGE-RAGE signaling pathway in diabetic complications	ame00270	2	0.0346
	Phototransduction—fly	ame04745	2	0.0346
	Longevity regulating pathway—multiple species	ame04213	2	0.0359
	Inositol phosphate metabolism	ame00562	2	0.0359
	Phosphatidylinositol signaling system	ame04070	2	0.0359
	Wnt signaling pathway	ame04310	2	0.0441
Fat body	Inositol phosphate metabolism	ame00562	3	0.0364
	Phosphatidylinositol signaling system	ame04070	3	0.0364
	Pentose and glucoronate interconversions	ame00040	2	0.0407
Flight muscle	Terpenoid backbone biosynthesis	ame00900	1	0.0299
	Phototransduction—fly	ame04745	1	0.0299

During the gene ontology (GO) analysis, we found that the GO term “Defense response to bacterium” was enriched in the fat body in all categories (Additional file 4: Table S2). We found no enrichment of GO terms in the brain or flight muscle.

## Discussion

The formation of a bee ball is a defensive strategy widely observed among honeybee species. However, balling by the Japanese honeybee, *A. cerana japonica*, is unique because of the precise temperature regulation and extreme heat production that are part of the process. To better understand the molecular basis of this behavior, we measured gene expression levels during bee ball formation in the brain, fat body, and flight muscle of worker honeybees using RNA-seq.

### Role of the rhodopsin signaling pathway in bee ball formation

A surprising result of this study was that several genes related to the rhodopsin signaling pathway were included in the DEGs of the “ball-only” group. The *rhodopsin long wavelength* gene and *arrestin 2* genes were upregulated during the balling in all three tissues. Three other genes related to rhodopsin signaling were also upregulated in the brain, namely *PLC*, *arrestin 1*, and *carotenoid isomerooxygenase*. Pathway analysis revealed that DEGs related to phototransduction were enriched in the brain and flight muscle, and those related to inositol phosphate metabolism and the phosphatidylinositol signaling, which often involve G-protein-coupled receptor (GPCR) signaling, were enriched in the brain and fat body.

Rhodopsin is primarily known as a photosensory protein involved in the downstream GPCR cascade with the transient receptor potential (TRP) channel family [42]. However, recent studies have found that rhodopsin also plays a role in thermal preference in *Drosophila* [7, 40, 61]. In animals, some of the TRP channel subfamilies (e.g., TRPV, TRPM, and TRPA) function as thermosensors [12]. In *Drosophila*, several channels of the TRPA group, such as TRPA1 and painless, act as heat sensors at the periphery, driving thermotaxis or avoidance of noxious heat [21, 36, 58, 61, 63]. For example, the preference of adult *D. melanogaster* toward temperatures of 24 °C is controlled by TRPA1, and its knockout results in defects in optimal temperature selection [21, 59]. In contrast, *Drosophila* larvae have a different temperature preference [36], which is under the control of several rhodopsin genes [61, 63]. Shen et al. [61] demonstrated that wild-type larvae preferred temperatures of 18 °C, whereas *Rh1* mutant larvae lost this preference. *Drosophila* individuals carrying mutations in a PLC gene (*norpA*), which is involved in the amplification of rhodopsin signals during phototransduction, also showed similar defects in thermotaxis [36, 61]. Sokabe et al. [63] showed that *Rh5* and *Rh6*, rather than *Rh1*, are required for the temperature preference toward 18 °C in late third-instar larvae. They also showed that *Rh5* and *Rh6* are co-expressed in *TRPA1*-neurons and function together with some molecules involved in Gq/PLC/TRP signaling cascade [63].

These studies consistently suggest that, in *Drosophila*, the thermal sensing function of rhodopsin is dependent on the G-protein signaling cascade and the TRPA1 channel being expressed in the same neurons. Although hymenopteran insects have lost TRPA1 homologs, they have acquired another gene in the TRP family called HsTRPA, which has a similar thermal and chemical sensing property as those of *Drosophila* TRPA1 [33]. The HsTRPA channel of *A. mellifera* (AmHsTRPA) is activated at around 34 °C [33], and might contribute to the maintenance of nest temperature. Therefore, it is possible that rhodopsin and its downstream G-protein pathway coordinate with HsTRPA to detect thermal stimuli in honeybees. Our RNA-seq data also confirmed that *AmHsTRPA* mRNA was present in all three tissues (Fig. 5).

*Arrestin 2*, another universally upregulated gene in the “ball-only” group, is known to interact with the GPCRs, such as the rhodopsin in animals [37, 73]. In the visual system of *Drosophila*, an excess of activated rhodopsin causes prolonged depolarized afterpotential (PDE), during which rhodopsin does not respond to new light stimuli. *Arr1* and *Arr2* of *Drosophila* desensitize rhodopsin and terminate the PDE [13]. If rhodopsin functions as a temperature sensor during bee balling, the role of *arrestin 2* might be to desensitize rhodopsin to ensure that it maintains its sensitivity to temperature changes.

**Fig. 6:**
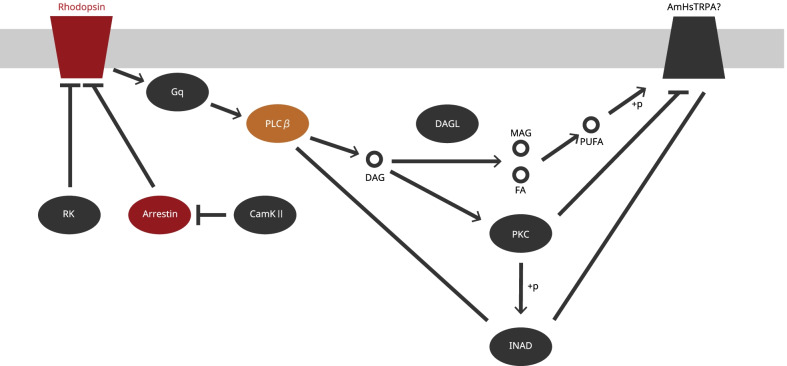
Model for the molecular mechanism of temperature sensing via rhodopsin signaling during *A. cerana japonica* balling behavior. Colored boxes indicate the genes involved in rhodopsin signaling (see “phototransduction—fly” in KEGG pathway) and expressed in all tissues in *A. cerana japonica* (see Additional file 1). Red boxes indicate the “ball-only” DEGs upregulated in all three tissues. Orange shows the “ball-only” DEG upregulated in the brain. Black indicates the genes expressed but not upregulated or downregulated in “ball-only” condition

Overall, our results raise the possibility that rhodopsin and its associated molecules function with HsTRPA during the detecttion of the thermal stimuli by *A. cerana japonica*. The putative molecular mechanism of temperature sensing via rhodopsin signaling during balling behavior is shown in Fig. 6. We confirmed the expression of multiple genes related to rhodopsin signaling in all tissues, although many of these genes were neither up- or downregulated during bee ball formation (Additional file 4: Table S3). We suggest that the upregulation of *rhodopsin* and *arrestin 2* alter the heat preference during the balling behavior to ensure that honeybees can continue monitoring the temperature that can sufficiently kill the attacker. In addition, *A. cerana japonica* workers are expected to make bee ball multiple times during a mass attack of *V. mandarinia*; therefore, changes in heat preference may be important for the formation and maintenance of multiple bee balls. Upregulation of *rhodopsin* and *arrestin 2* in all tissues is also consistent with a previous report that adult honeybees were capable of sensing temperatures throughout their body [28]. However, our results are not entirely consistent; we observed the upregulation of other genes in rhodopsin signaling (e.g., *PLC* and *arrestin 1*) only in the brain and not in the fat body and flight muscle. This result may suggest that different sets of molecules are involved in the thermosensory rhodopsin pathway in other tissues. At present, we cannot entirely rule out the possibility that these molecules function in processes other than temperature sensing. Further study is warranted to elucidate the role of rhodopsin signaling during bee ball formation.

### DEGs in the brain

Our results showed that the expression of genes related to behavior, inflammation, metabolism, and visual signals changed in the brain during balling behavior. Dopamine influences locomotor activity in invertebrates [9, 14], and it affects the behavioral activity in honeybees [22, 44]. In the present study, expression of *NR4A2* increased and *NRP1* decreased in the “ball-only” condition; in previous studies, *NR4A2* and *NRP1* affected dopamine levels in the brains of mice and rats, respectively [16, 45]. Therefore, these genes could be involved in some actions during balling behavior via the regulation of dopamine levels. Moreover, Hunt et al. [27] found that *arrestin 1* was included in the QTL related to stinging behavior, and that its expression level was higher in stinging *A. mellifera* relative to non-stinging young *A. mellifera*. Honeybees often sting the hornet during the bee ball formation [20, 50, 77]. *A. cerana japonica* was previously reported not to sting during the balling [50], but a recent study found frequent stinging by *A. cerana cerana* during the ball formation [20]. Therefore, the upregulation of *arrestin 1* may induce the stinging behavior or enhance an alternative signal that acts specifically on attacking behaviors in *A. cerana japonica*. Amino acid sequence comparison of Arrestin 1 between *A. cerana japonica* and *A. mellifera* revealed that some of the amino acid mutations between the two species were deleterious (Additional file 6: Table S4). The difference in the amino acid sequence of arrestin 1 between these species could be related to their behavioral difference during balling.

Inflammation is a reaction caused by injury or infection with several symptoms, including rashes, fever, swelling, and pain [57]. Prostaglandins are bioactive substances involved in inflammation [18, 57]. We observed the upregulation of prostaglandins in the “ball-only” condition [78], indicating that HPGD is involved in the suppression of inflammation caused by the high-temperature experienced during balling.

Carbohydrates are required for brain activity in many animals [38]. In the current study, expression of the genes encoding malate dehydrogenase (MDH) and NADH dehydrogenase subunit 5, which are involved in sugar metabolism, increased in the “ball-only” condition. MDH is involved in the Krebs cycle [46], while NADH dehydrogenase is involved in mitochondrial electron transport [43]. Glucose metabolism reportedly increases during long-term memory formation in mushroom bodies in *Drosophila* [56], suggesting that carbohydrates are used for higher-order functions in the insect brain. In *A. cerana japonica*, higher information processing in mushroom body is reportedly activated during bee balling [71]; therefore, the upregulation of MDH- and NADH-related genes could promote sugar metabolism in the brain of workers during balling behavior.

### DEGs in fat body

Fat bodies are insect organs that store and transport energy substrates such as carbohydrates [6]. Since honeybees mainly use carbohydrates during high-energy metabolic processes, such as flight and heat production [52], the fat body likely plays a vital role in during the formation of hot defensive bee ball. Our results showed that the expression levels of genes related to energy metabolism, stress tolerance, and immunity were altered in the fat body during balling.

Of the “ball-only” DEGs involved in energy metabolism, the expression of three *Tret1* genes increased, whereas that of acetyl-coA carboxylase decreased during balling. Insects use trehalose, generated from glycogen in the fat body, as a major hemolymph sugar [75]. In *Polypedilum vanderplanki*, Tret1 transports trehalose, synthesized in the fat body, into the hemolymph [30]. Therefore, these genes are expected to be used to transport carbohydrates for energy metabolism in the flight muscle for heat production during balling. *Acetyl-coA carboxylase* is a gene involved in fatty acid synthesis [43]. During balling behavior, fatty acid synthesis may be suppressed because carbohydrates are instead required for heat production.

Immunity is an energetically costly function in insects [4, 17]. In *A. mellifera*, for example, the number of foraging flights was reduced in immune-activated workers [3]. Our GO analysis revealed that “defense response to bacterium” was enriched in the fat body. This GO term is associated with *abaecin-like hymenoptaecin* and *defensin-1*, which were all downregulated in the “ball-only” condition, indicating that immunity may be suppressed during bee ball formation because energy needs to be allocated for metabolism.

High temperatures are a major stressor for insects. In *A. cerana japonica*, the survival rate of workers that participate in balling is reduced [77], suggesting that the high temperature in the bee ball affects the honeybee workers as well as the hornet. We found that gene expression of three heat shock proteins, *HSP90, HSc70*, and *HSP97,* increased in the “ball-only” condition. Such heat shock proteins are involved in response to temperature stresses in insects [11, 31, 34, 62, 68, 70]; thus, the aforementioned HSP genes may function in heat tolerance during balling behavior.

### DEGs in flight muscle

Flight muscles are the primary heat-generating organs in insects [25]. Although we did not identify strong candidate genes that enabled extraordinary heat production in the flight muscle of *A. cerana japonica*, we were able to detect several “ball-only” DEGs related to the exoskeleton. Insect flight muscles adhere to the thorax exoskeleton, which is composed of chitin, cuticle protein, phenols, and lipids [46]. We found that the expression of genes encoding endochitinase A1 increased, while those of cuticle protein genes decreased. Chitinase is used to degrade chitin in insects [35, 46]; chitin is a polymer of N-acetylglucosamine, which is the raw material of trehalose. Thus, our results may imply that honeybees produce additional sugar for energy metabolism by digesting their exoskeleton during bee ball formation. Additionally, these genes may influence the contraction of flight muscles by regulating the strength of the exoskeleton.

## Conclusion

To detect the candidate genes underlying the defensive balling behavior of *A. cerana japonica*, we conducted extensive gene expression analysis. Intriguingly, we found that the expression level of several genes involved in rhodopsin signaling increased in the brain, fat body, and flight muscle during the balling behavior. Our results also revealed expression changes in genes related to energy metabolism and heat-stress response. These results will provide a new perspective on the specific defense behavior of Japanese honeybees.

## Methods

### Bee sampling

We conducted a series of experiments to induce bee ball formation in *A. cerana japonica* and collected samples for RNA extraction. We used a colony of *A. cerana japonica* from Nagano Prefecture that was reared at the apiary of Tamagawa University in Tokyo. Experiments were performed in the autumn of 2015. A *Vespa mandarinia* worker used in the experiment was captured on the campus of Tamagawa University in Tokyo.

Bees were sampled for RNA extraction at several different stages of the experiment; these samples were later used for comparison of gene expression. The experimental outline is shown in Fig. 2.

Worker honeybees show age-dependent division of labor. Therefore, the following process was undertaken to collect 15-day-old bees (known to participate in bee balling [77]; from the nest and the bee ball, the following operation was performed. A brood comb containing pupae was collected from a hive and reared in an incubator at 34 °C. Newly emerged bees were marked on the thorax using colored paint markers (PX-21; Mitsubishi Pencil, Japan) and returned to the hive. The balling experiment was conducted when the marked worker bees were 15 days old. Before the experiment, the marked workers were collected from the colony. Some of the workers were immediately anesthetized on ice water for tissue dissection (denoted “before,” n = 3), and the remaining bees were placed in plastic cups with 1 mol/L sucrose solution and incubated overnight at 31 °C in the dark. The incubated bees underwent further incubation, either for an additional 30 min at 31 °C in the dark (denoted “normal”, n = 3), or for 30 min at 46 °C in the dark (denoted “heated,” n = 4). After the additional 30-min incubation period, workers were collected and anesthetized on ice water. In the following analysis, “before” and “normal” were treated as “control” group to improve the statistical power; this was appropriate because two groups were placed under the essentially same environmental conditions.

For the balling experiment, we used a single *V*. *mandarinia* worker with its stinger removed to induce bee ball formation in *A. cerana japonica*. The hornet was hung from a copper wire and presented at the entrance of the hive. Soon after the ball formation, it was pulled away from the nest. The bee ball was maintained for 30 min. Once the ball had dissipated, worker honeybees that had participated in the balling were collected using a long tweezer and anesthetized on ice water for tissue dissection (denoted “balling,” n = 4).

### RNA extraction

Brain, flight muscle, and fat body were dissected from sampled bees (n = 4, 4, and 6 in balling, heated, and control groups, respectively). After tissue homogenization, the total RNA of the brain and fat body was extracted using RNeasy Micro Kit (Qiagen) and those of flight muscle were extracted using RNeasy Fibrous Tissue Mini Kit (Qiagen) following the manufacturer’s protocol. Total RNA samples were stored at − 80 °C until library construction.

### RNA-seq data analysis

Library constructions from RNA samples and sequences were conducted at Beijing Genomics Institute (BGI, Shenzhen, China). All libraries were constructed using an Illumina HiSeq 4000 paired-end sequencer. The low-quality raw RNA-seq reads were filtered using the fastx_clipper of FASTX Toolkit version 0.0.13. To reconstruct transcripts, reads derived from 42 samples (3 organs of 14 individuals) were assembled using Trinity version 2.1.1 [19]. Adapter sequences were removed during this process using the Trimmomatic tool in Trinity.

We performed reciprocal BLAST searches to annotate orthologous pairs between *A. cerana japonica* and *A. mellifera*. We first downloaded the amino acid sequences of the whole genome of *A. mellifera* from Ensembl Metazoa release 37. We then performed TBLASTN searches (e-value = 0.00001) using the amino acid sequences of *A. mellifera* as input queries and *A. cerana japonica* contigs as databases. Subsequently, we extracted *A. cerana japonica* contigs with the highest match to each *A. mellifera* gene. BLASTX (e-value = 0.00001) was conducted using the selected set of *A. cerana japonica* contigs as input queries and *A. mellifera* protein sequences as databases. These processes allowed us to obtain a set of 10,172 one-to-one orthologous pairs between *A. cerana japonica* contigs and *A. mellifera* proteins.

### Calculating DEGs

We mapped the reads from each RNA-seq sample onto the de novo transcripts, and calculated the read count and FPKM value for each transcript using RSEM version 1.2.30. PCA analysis was performed with FPKM values using “prcomp” function in R version 3.3.3. Differentially expressed genes (DEGs) were estimated using the TCC package (FDR < 0.05) in edgeR [67]. To detect the genes that were putatively associated with balling behavior, DEGs were identified in the following comparisons: (1) “balling” vs. “control” (Comparison 1) and (2) “control” vs. “heated” (Comparison 2). The DEGs in Comparison 1 were expected to include not only the genes regulating the bee ball formation but also those functioning in thermal tolerance, whereas the DEGs in Comparison 2 were expected to include genes for thermal tolerance during high-temperature exposure. By removing the DEGs that overlapped between Comparisons 1 and 2 from those of Comparison 1, we narrowed down the DEGs for which expression was altered specifically during the bee ball formation, we denoted these genes as “ball-only” DEGs. The identities of DEGs were estimated by the BLAST search using the NCBI non-redundant database.

### Pathway analysis and GO analysis

To detect the functional biases of DEGs in Comparison 1, Comparison 2, and “ball-only”, we performed the Kyoto Encyclopedia of Genes and Genomes (KEGG) pathway analysis using KOBAS version 3.0. P values were corrected using the Benjamin-Hochberg correction [8], and terms with a corrected P-value of less than 0.05 were considered significantly enriched terms.

To detect the DEGs’ functional biases in Comparison 1, Comparison 2, and “ball-only”, we performed gene ontology GO analysis using the goseq package in R version 3.3.3. P values were corrected using the Benjamin-Hochberg correction, and terms with a corrected P-value of less than 0.05 were considered significantly enriched terms.

## Supplementary Information

**Additional file 1.** RNA-seq count data obtained in the current study. Under three conditions of balling, heated, and control, the gene expression levels of 3 tissues of brain, fat body, and flight muscle were measured by RNA-seq. The count data for each condition and tissue are shown in separate worksheets.

**Additional file 2.** RNA-seq FPKM data obtained in the current study. Under three conditions of balling, heated, and control, the gene expression levels of 3 tissues of brain, fat body, and flight muscle were measured by RNA-seq. The FPKM data for each condition and tissue are shown in separate worksheets.

**Additional file 3.** Defferentially expressed genes (DEGs) detected in the comparison1 and comparison 2. DEGs were listed in rank order based on the Trinity_ID. Red and blue letters indicate whether the gene expression level is upregulated or downregulated with relative to control.

**Additional file 4.** Supplementary Tables 1 to 4.

**Additional file 5: Figure S1.** Profiles of the FPKM values in the RNA-seq data. (a) FPKM density distribution of three groups in each tissue. (b) FPKM distribution of three groups in each tissue.

**Additional file 6: Figure S2.** M-A plots of DEGs between every two groups. (a) M-A plots of DEGs in “balling” vs. “control” in each tissue. (b) M-A plots of DEGs in “heated” vs. “control” in each tissue. Pink points indicate the differentially expressed genes.

**Additional file 7: Figure S3.** Number of DEGs in each tissue. a) DEGs in “Comparison 1” (“balling” vs. “control”). b) DEGs in “Comparison 2” (“heated” vs. “control”).

## Data Availability

RNA-seq raw sequence reads are available through the DDBJ Sequence Read Archive (https://ddbj.nig.ac.jp) under accession no. PRJDB10552. The RNA-seq count data used in the differential gene expression analysis are listed in Additional file 1 (Supporting information). FPKM values used in the principal components analysis are listed in Additional file 2 (Supporting information). A complete list of DEGs is provided in Additional file 3. R scripts used herein are available upon request.

## Abbreviations

DEGs: Differentially expressed genes
GO: Gene ontology
