# Supplementary material for: Genes associated with hot defensive bee ball in the Japanese honeybee, Apis cerana japonica
Source: BMC Ecol Evol. 2022 Mar 16;22:31. doi: 10.1186/s12862-022-01989-9 (PMC8925055; doi:10.1186/s12862-022-01989-9)
Supplement: Supplementary file 4 — Additional file 4. Supplementary Tables 1 to 4. [file 12862_2022_1989_MOESM4_ESM.docx]

**Supplementary Tables**

**Table S1**. Result of the GO analysis in “Comparison1” (“balling: bees participated in balling for 30 minutes” vs. “control: bees incubated 30 minutes in 31 °C or captured before balling formation”), “Comparison2” (“heated: bees incubated 30 minutes in 46 °C” vs. “control”), and “ball-only” (A group of DEGs which removed overlapped DEGs between Comparison 1 and Comparison 2 from those of Comparison 1).

| Comparison | Organ | GO term | GO term | Number of Genes | Over represented  P-value |
| --- | --- | --- | --- | --- | --- |
|  |  |  | accesion number |  |  |
| balling vs. control (Comparison1) | Fat body | Defense response to bacterium | GO:0042742 | 3 | 5.80E-06 |
| heated vs. control (Comparison2) | Fat body | Defense response to bacterium | GO:0042742 | 3 | 4.85E-06 |
|  | Fat body | Innate immune response | GO:0045087 | 3 | 1.40E-05 |
| ball-only | Fat body | Defense response to bacterium | GO:0042742 | 3 | 2.84E-06 |

**Table S2**. Result of pathway analysis in “Comparison1” and “Comparison2”.

| Comparison | Organ | KEGG pathway | ID | Number of Genes | Corrected P-value |
| --- | --- | --- | --- | --- | --- |
| ‘balling’ vs ‘control’ | Brain | Longevity regulating pathway - multiple species | ame04213 | 8 | 6.18E-09 |
| (Comparison1) |  | Protein processing in endoplasmic reticulum | ame04141 | 8 | 6.46E-06 |
|  |  | Glycerophospholipid metabolism | ame00564 | 3 | 0.034536319 |
|  | Fat body | Protein processing in endoplasmic reticulum | ame04141 | 10 | 7.51E-07 |
|  |  | Longevity regulating pathway - multiple species | ame04213 | 7 | 7.51E-07 |
|  |  | Inositol phosphate metabolism | ame00562 | 3 | 0.043637259 |
|  |  | Phosphatidylinositol signaling system | ame04070 | 3 | 0.043637259 |
|  |  | Pentose and glucuronate interconversions | ame00040 | 2 | 0.043637259 |
|  |  | Spliceosome | ame03040 | 4 | 0.043637259 |
|  | Flight muscle | Protein processing in endoplasmic reticulum | ame04141 | 3 | 0.001647774 |
|  |  | Longevity regulating pathway - multiple species | ame04213 | 4 | 0.003119414 |
| ‘heated’ vs ‘control’ | Brain | Longevity regulating pathway - multiple species | ame04213 | 6 | 1.51E-10 |
| (Comparison2) |  | Protein processing in endoplasmic reticulum | ame04141 | 8 | 2.50E-05 |
|  |  | Spliceosome | ame03040 | 5 | 0.0064593 |
|  |  | Glycerophospholipid metabolism | ame00564 | 3 | 0.037346478 |
|  | Fat body | Longevity regulating pathway - multiple species | ame04213 | 9 | 1.88E+08 |
|  |  | Protein processing in endoplasmic reticulum | ame04141 | 7 | 0.002464758 |
|  |  | Glycerophospholipid metabolism | ame00564 | 5 | 0.002513442 |
|  | Flight muscle | Longevity regulating pathway - multiple species | ame04213 | 5 | 0.000280734 |
|  |  | Protein processing in endoplasmic reticulum | ame04141 | 6 | 0.001952549 |

**Table S3**. Result of the provean in arrestin1.

| Position | *A. c. japonica* | *A. mellifera* | Provean | mutation |
| --- | --- | --- | --- | --- |
| 45 | D | G | -4.229 | Deleterious |
| 91 | I | V | 0.364 | Neutral |
| 113 | K | E | 0.191 | Neutral |
| 136 | V | I | 2.4 | Neutral |
| 168 | A | T | 0.957 | Neutral |
| 300 | V | I | -2.345 | Neutral |
| 360 | E | D | -0.647 | Neutral |
| 362 | V | L | -0.038 | Neutral |
| 365 | A | V | -0.334 | Neutral |

**Table S4**. Count data of genes included in the rhodopsin signaling (see “phototransduction - fly” in KEGG pathway) and AmHsTRPA. Bold letters indicate genes whose expression is changed only in "ball-only".

| Organ | ID | Gene | Average number of read count | | |
| --- | --- | --- | --- | --- | --- |
|  |  |  | Balling | Control | Heated |
| Brain | TRINITY_DN44852_c0_g1_i2 | rhodopsin, long-wavelength | 16086.47 | 6237.26 | 7679.94 |
|  | TRINITY_DN46710_c0_g2_i1 | arrestin homolog | 14845.39 | 6080.90 | 8052.73 |
|  | TRINITY_DN51466_c0_g1_i1 | 1-phosphatidylinositol 4,5-bisphosphate phosphodiesterase-like | 481.75 | 146.33 | 139.00 |
|  | TRINITY_DN57804_c1_g2_i1 | arrestin homolog | 805.25 | 243.17 | 246.00 |
|  | TRINITY_DN65593_c0_g1_i1 | carotenoid isomerooxygenase | 702.61 | 154.36 | 285.81 |
|  | TRINITY_DN63928_c0_g1_i1 | G protein alpha q subunit isoform X5 | 2030.75 | 2567.17 | 2173.25 |
|  | TRINITY_DN65403_c2_g1_i2 | sn1-specific diacylglycerol lipase alpha isoform X1 | 286.75 | 507.73 | 358.80 |
|  | TRINITY_DN66747_c5_g1_i1 | protein kinase C | 12958.62 | 15166.79 | 13496.62 |
|  | TRINITY_DN65750_c14_g1_i1 | G protein-coupled receptor kinase 1 isoform X1 | 3207.50 | 4339.83 | 3948.50 |
|  | TRINITY_DN66435_c3_g3_i12 | calcium/calmodulin-dependent protein kinase II | 324.49 | 513.72 | 558.40 |
|  | TRINITY_DN64854_c2_g1_i8 | inaD-like protein | 1262.46 | 1443.77 | 828.81 |
|  | TRINITY_DN49086_c0_g2_i2 | AmHsTRPA | 599.51 | 524.54 | 522.14 |
|  |  |  |  |  |  |
|  |  |  |  |  |  |
| Fat body | TRINITY_DN44852_c0_g1_i2 | rhodopsin, long-wavelength | 335.62 | 78.16 | 124.24 |
|  | TRINITY_DN46710_c0_g2_i1 | arrestin homolog | 261.89 | 85.85 | 125.69 |
|  | TRINITY_DN51466_c0_g1_i1 | 1-phosphatidylinositol 4,5-bisphosphate phosphodiesterase-like | 13.50 | 5.83 | 6.50 |
|  | TRINITY_DN57804_c1_g2_i1 | arrestin homolog | 38.00 | 14.67 | 24.75 |
|  | TRINITY_DN65593_c0_g1_i1 | carotenoid isomerooxygenase | 213.69 | 94.39 | 106.60 |
|  | TRINITY_DN63928_c0_g1_i1 | G protein alpha q subunit isoform X5 | 741.75 | 656.83 | 583.50 |
|  | TRINITY_DN65403_c2_g1_i2 | sn1-specific diacylglycerol lipase alpha isoform X1 | 41.93 | 40.98 | 26.65 |
|  | TRINITY_DN66747_c5_g1_i1 | protein kinase C | 603.00 | 495.50 | 688.50 |
|  | TRINITY_DN65750_c14_g1_i1 | G protein-coupled receptor kinase 1 isoform X1 | 1742.00 | 1340.67 | 1093.25 |
|  | TRINITY_DN66435_c3_g3_i12 | calcium/calmodulin-dependent protein kinase II | 32.47 | 1.91 | 29.06 |
|  | TRINITY_DN64854_c2_g1_i8 | inaD-like protein | 125.33 | 135.06 | 129.44 |
|  | TRINITY_DN49086_c0_g2_i2 | AmHsTRPA | 67.38 | 39.92 | 36.32 |
|  |  |  |  |  |  |
|  |  |  |  |  |  |
| Flight muscle | TRINITY_DN44852_c0_g1_i2 | rhodopsin, long-wavelength | 578.61 | 89.83 | 196.98 |
|  | TRINITY_DN46710_c0_g2_i1 | arrestin homolog | 447.67 | 128.69 | 189.32 |
|  | TRINITY_DN51466_c0_g1_i1 | 1-phosphatidylinositol 4,5-bisphosphate phosphodiesterase-like | 21.75 | 3.83 | 8.25 |
|  | TRINITY_DN57804_c1_g2_i1 | arrestin homolog | 73.25 | 18.33 | 27.00 |
|  | TRINITY_DN65593_c0_g1_i1 | carotenoid isomerooxygenase | 156.70 | 204.23 | 178.23 |
|  | TRINITY_DN63928_c0_g1_i1 | G protein alpha q subunit isoform X5 | 336.50 | 326.33 | 336.25 |
|  | TRINITY_DN65403_c2_g1_i2 | sn1-specific diacylglycerol lipase alpha isoform X1 | 21.03 | 37.40 | 21.68 |
|  | TRINITY_DN66747_c5_g1_i1 | protein kinase C | 939.99 | 956.00 | 885.75 |
|  | TRINITY_DN65750_c14_g1_i1 | G protein-coupled receptor kinase 1 isoform X1 | 3300.00 | 2879.00 | 2696.50 |
|  | TRINITY_DN66435_c3_g3_i12 | calcium/calmodulin-dependent protein kinase II | 23.33 | 20.78 | 105.37 |
|  | TRINITY_DN64854_c2_g1_i8 | inaD-like protein | 108.11 | 214.90 | 162.33 |
|  | TRINITY_DN49086_c0_g2_i2 | AmHsTRPA | 38.80 | 59.92 | 22.60 |
