# Supplementary material for: Genes associated with hot defensive bee ball in the Japanese honeybee, Apis cerana japonica
Source: BMC Ecol Evol. 2022 Mar 16;22:31. doi: 10.1186/s12862-022-01989-9 (PMC8925055; doi:10.1186/s12862-022-01989-9)
Supplement: Supplementary file 5 — Additional file 5: Figure S1. Profiles of the FPKM values in the RNA-seq data. (a) FPKM density distribution of three groups in each tissue. (b) FPKM distribution of three groups in each tissue. [file 12862_2022_1989_MOESM5_ESM.pdf]

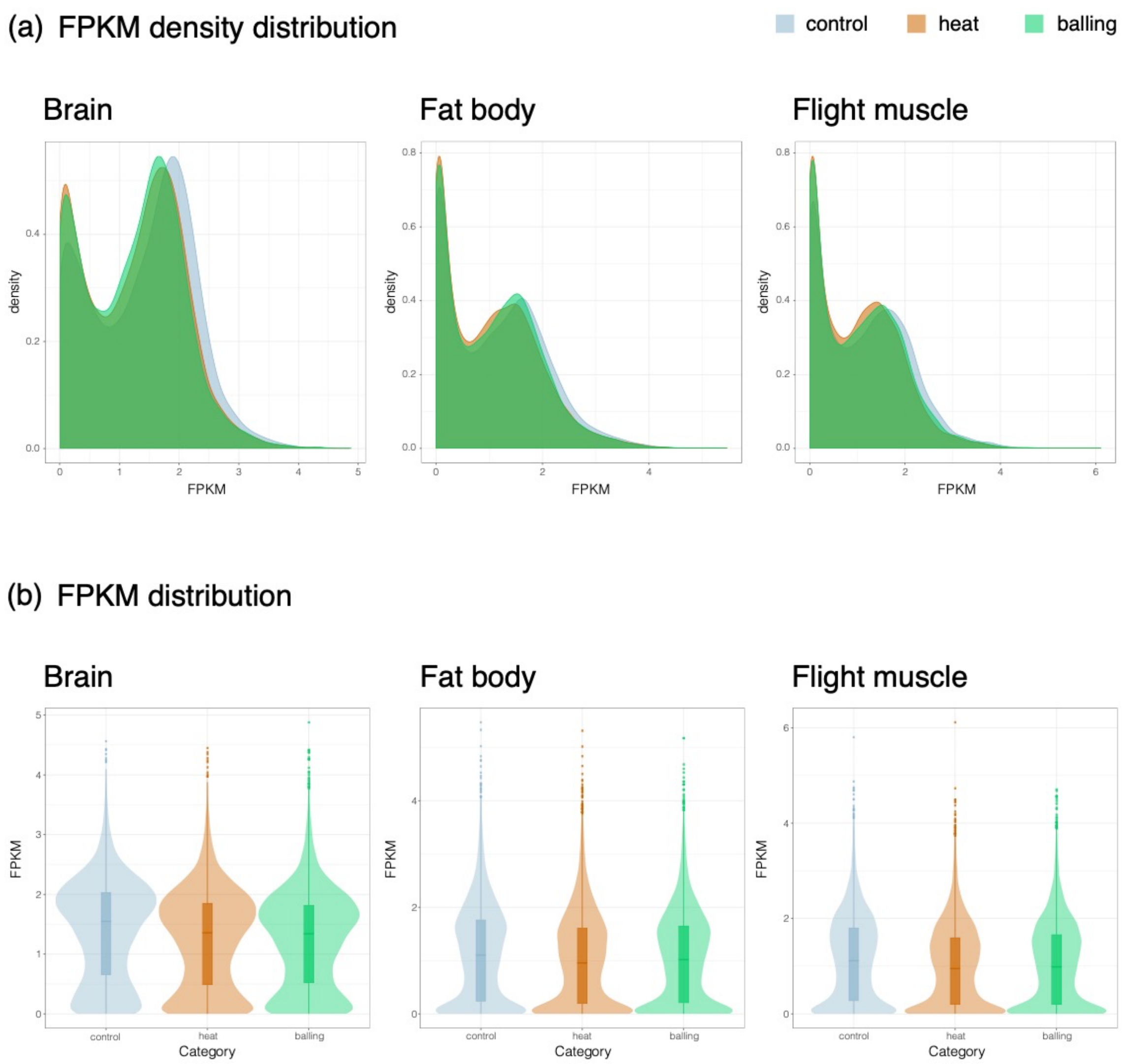

Figure S1 Profiles of the FPKM values in the RNA-seq data. (a) FPKM density distribution of three groups in each tissue. (b) FPKM distribution of three groups in each tissue.
