# Supplementary material for: Genes associated with hot defensive bee ball in the Japanese honeybee, Apis cerana japonica
Source: BMC Ecol Evol. 2022 Mar 16;22:31. doi: 10.1186/s12862-022-01989-9 (PMC8925055; doi:10.1186/s12862-022-01989-9)
Supplement: Supplementary file 6 — Additional file 6: Figure S2. M-A plots of DEGs between every two groups. (a) M-A plots of DEGs in “balling” vs. “control” in each tissue. (b) M-A plots of DEGs in “heated” vs. “control” in each tissue. Pink points indicate the differentially expressed genes. [file 12862_2022_1989_MOESM6_ESM.pdf]

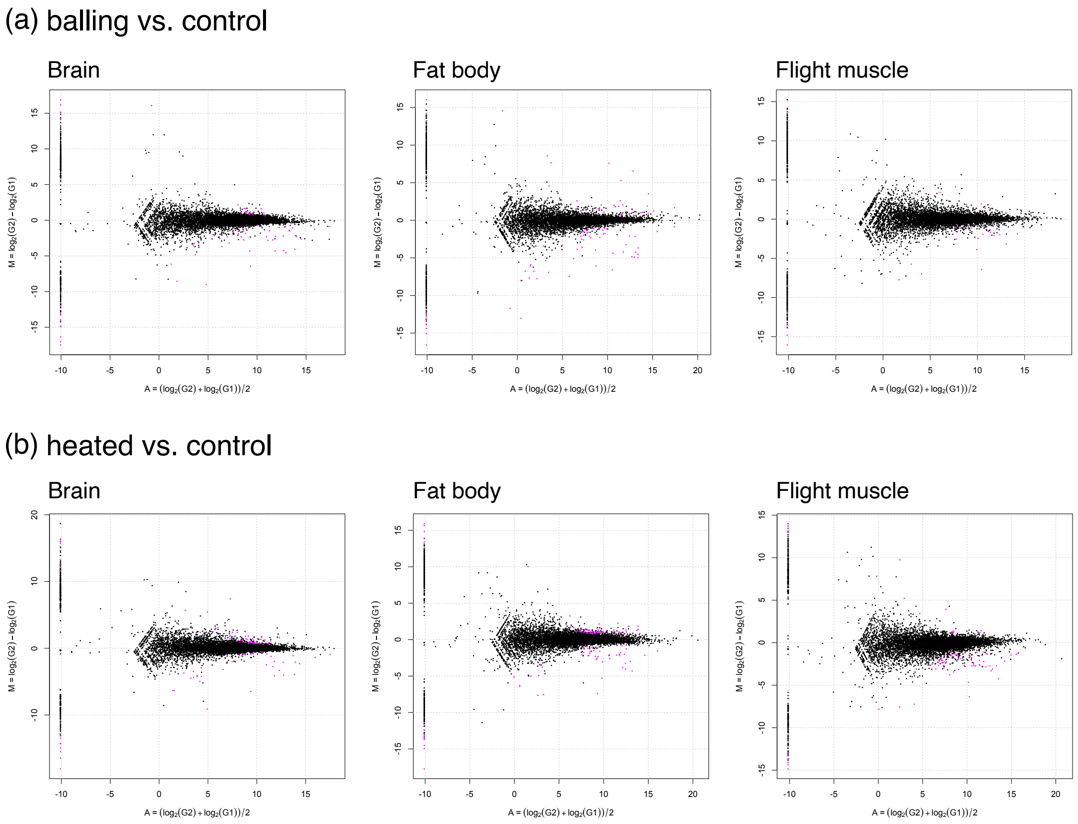

Figure S2 M-A plots of DEGs between every two groups. (a) M-A plots of DEGs in "balling" vs. "control" in each tissue. (b) M-A plots of DEGs in "heated" vs. "control" in each tissue. Pink points indicate the differentially expressed genes.
