# Supplementary material for: Genes associated with hot defensive bee ball in the Japanese honeybee, Apis cerana japonica
Source: BMC Ecol Evol. 2022 Mar 16;22:31. doi: 10.1186/s12862-022-01989-9 (PMC8925055; doi:10.1186/s12862-022-01989-9)
Supplement: Supplementary file 7 — Additional file 7: Figure S3. Number of DEGs in each tissue. a) DEGs in “Comparison 1” (“balling” vs. “control”). b) DEGs in “Comparison 2” (“heated” vs. “control”). [file 12862_2022_1989_MOESM7_ESM.pdf]

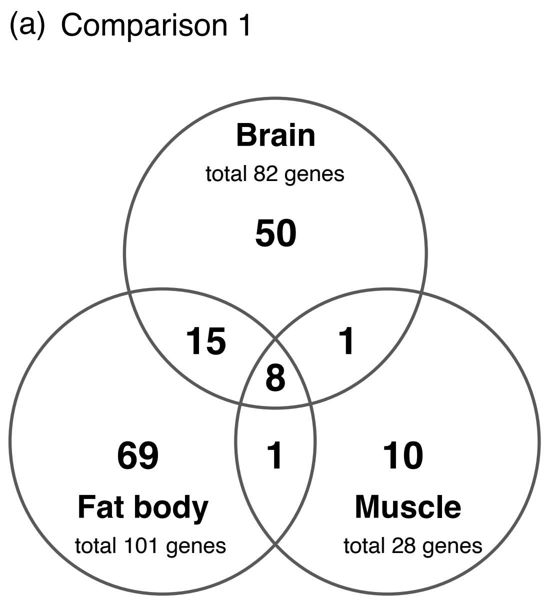

Figure S3. Number of DEGs in each tissue. a) DEGs in "Comparison 1" ("balling" vs. "control"). b) DEGs in "Comparison 2" ("heated" vs. "control").
